# Scientific publishing during the COVID-19 pandemic

**DOI:** 10.1007/s00234-020-02608-4

**Published:** 2020-11-20

**Authors:** Rüdiger von Kummer

**Affiliations:** Institute and Policlinic of Diagnostic and Interventional Neuroradiology, Universitätsklinikum Dresden, Dresden, Germany

While celebrating *Neuroradiology’s* 50th anniversary in early 2020 (https://www.springer.com/journal/234/updates/17776350), our Editors did not envisage how the global pandemic spread of acute respiratory syndrome coronavirus 2 with severe Coronavirus Disease-2019 (COVID-19) and its concomitant rise of victims since March would impact scientific publishing. Interestingly, when compared to the average monthly rate of submissions in 2019, we observed an almost 60% increase of monthly submissions starting in March 2020. We accelerated the peer-review process by asking for prompt reviews of COVID-19-related articles, were as flexible as possible regarding timelines, and sought articles providing novel neuroimaging aspects of COVID-19. We were happy to publish “COVID-19: A primer for Neuroradiologists” in April [1]. August followed with a pictoral review of “Bilateral lesions of the basal ganglia and thalami (central grey matter)” that included COVID-19-associated acute necrotizing encephalopathy [2]. Although *Neuroradiology* publishes only exceptional case reports, we presented in September a patient with “COVID-19-induced anosmia associated with olfactory bulb atrophy,” who had a pre-COVID MRI for comparison [3]. This report supports the view that the virus may infect olfactory epithelial support cells. Finally, in October, we published another case report, “Acute disseminated encephalomyelitis in a COVID-19 pediatric patient,” which clearly illustrates the spectrum of immune reaction of the central nervous system to the virus [4].

In addition to these remarkable articles, *Neuroradiology* published 143 original studies, 18 state-of-the-art reviews, six Editorials, two guideline papers, and 13 Letters to the Editor in 2020. I thank our authors, reviewers, editors, the members of the Editorial Board, and Springer’s technical team for contributing to the quality of published articles and maintaining high publication ethics standards. My special thanks go to those members who have served for six or more years on our Editorial Board who will now retire to create space for new members.

There is no doubt that the pandemic will continue in 2021. I am confident, however, that vaccination will soon protect those at high risk while new medications and therapies will help prevent poor outcomes. This optimistic view is justified as long as scientists continue to communicate their findings and insights quickly and without restrictions. Ultimately, it is our job to facilitate wide dissemination of evidence-based scientific truths, and together, we will continue to do so.
